# ESR Bridges: new developments in imaging and treatment of the unknown primary squamous cell carcinoma of the head and neck—a multidisciplinary view

**DOI:** 10.1007/s00330-025-11977-4

**Published:** 2025-09-16

**Authors:** Minerva Becker, Michiel van den Brekel, Roberto Maroldi

**Affiliations:** 1 Unit of Head and Neck and Maxillo-facial Radiology, Division of Radiology, Diagnostic Department, Geneva University Hospitals, University of Geneva, Geneva, Switzerland; 2 Department of Head and Neck Oncology and Surgery, Netherlands Cancer Institute–Antoni van Leeuwenhoek, Amsterdam, The Netherlands; 3 Radiological Sciences, DSMC Department, University of Brescia, Brescia, Italy

## What is the clinical need to be addressed?

Head and neck squamous cell carcinoma of unknown primary (HNSCCUP) accounts for 2–5% of all head and neck (HN) cancers. It is characterised by cytologically or histologically confirmed metastatic lymph nodes in the neck without identification of a primary tumour despite a thorough clinical, radiologic and histologic workup [1–3]. Although published articles do not always specify whether reported data refer to “clinically occult”, “radiologically occult” or “histologically occult” tumours, only tumours undetected after all investigations should be considered as true HNSCCUP [2, 3]. As treatment modalities—surgery, radiotherapy, and systemic therapies—are tailored according to the anatomical tumour origin, precise detection of the unknown primary is paramount to improving prognosis, preserving function, and reducing treatment-related toxicity [1].

Initial patient evaluation comprises a detailed history focusing on symptoms like dysphagia and hoarseness, and risk factors such as tobacco, alcohol, sexual behaviour, and prior malignancies. The nodal metastasis location also guides primary tumour site suspicion, e.g., levels II-III indicating oropharyngeal cancers, level Va suggesting nasopharyngeal or skin malignancies, and levels IV, VI and Vb pointing at primaries of thyroid and skin origin or outside the neck. Furthermore, some cancers, e.g., high-risk Human Papilloma Virus (HPV)-related oropharyngeal squamous cell carcinoma (SCC) are known for early lymphatic metastases despite small or undetectable primary tumours. Initial physical examination includes palpation and flexible fibreoptic endoscopy, often enhanced by narrow band imaging (NBI). NBI increases the detection rate of malignant mucosal lesions by highlighting mucosal neo-angiogenesis using blue and green light wavelengths.

Pathologic SCC confirmation typically begins with ultrasound-guided fine-needle aspiration cytology (USFNAC) of the enlarged lymph node, followed by cross-sectional imaging and panendoscopy under general anaesthesia with biopsy of suspicious mucosal sites. Due to low diagnostic yield, traditional random biopsies are no longer proposed. When directed biopsies fail to locate the primary tumour, tonsillectomy and base of tongue mucosectomy are carried out, as these sites are the most common origins of occult primary tumours, accounting for roughly 45% of cases [4]. Minimally invasive techniques—transoral robotic surgery (TORS) and transoral laser microsurgery (TLM)—are effective diagnostic tools, especially when prior clinical, radiological, and panendoscopic evaluations are negative (40% of cases), with notably higher detection rates in HPV-positive HNSCCUP [2].

## Opportunities and challenges—radiologist’s view

Imaging is crucial in the diagnostic pathway of HNSCCUP, guiding biopsy sites, surgical planning, and radiotherapy target delineation. US, as a first-line imaging modality, confirms that the neck lump is caused by lymphadenopathy. It precisely indicates the involved neck levels, whether nodal involvement is unilateral or bilateral, and whether there is involvement of a single versus multiple nodes. Moreover, US highlights imaging findings suggesting HPV-positivity, e.g., (pseudo)cystic nodes. USFNAC, favoured for its safety and cost-effectiveness, has a high diagnostic reliability for SCC with sensitivity around 90% and specificity exceeding 95% [5]. As high-risk HPV is implicated in most occult oropharyngeal cancers with levels II/III nodal metastases, p16 immunohistochemistry testing on the aspirate, followed by PCR-based DNA or RNA in situ hybridisation help localise the primary tumour [6]. Epstein-Barr virus (EBV) testing via EBER in situ hybridisation is recommended for HPV-negative cases, as EBV is strongly associated with nasopharyngeal carcinoma. Nevertheless, negative EBER does not exclude the nasopharynx as tumour origin, especially in non-endemic regions. Determining the HPV and EBV status is important for primary tumour localisation, as key prognostic and therapeutic factors in HNSCCUP management and also for AJCC/UICC TNM classification [2, 7]. According to the 9th AJCC/UICC TNM classification, if the biopsied lymph node is EBV positive, the nasopharyngeal classification is applied, and if the node is HPV and/or p16 positive, the p16-positive oropharyngeal classification is used [7]. US-guided core needle biopsy (CNB) serves as a complementary technique in inconclusive FNAC cases and for the assessment of additional biomarkers, e.g., Programmed Death-Ligand 1 (PD-L1) to guide immunotherapy and personalised treatment or a UV-signature at DNA sequencing to confirm skin origin.

Multiparametric MRI (with DWI and contrast-enhanced sequences) from the skull base to the thoracic inlet is the recommended first imaging modality in HNSCCUP [3]. Contrast-enhanced CT (CECT) is also used initially in many institutions due to its wide availability, rapid image acquisition, and relatively low cost compared to MRI and PET CT. However, multiparametric MRI offers superior tissue characterisation, especially in small oropharyngeal and nasopharyngeal tumours, the reported detection rate for HNSCCUP being around 40% [8]. The role of MRI and CECT in the context of HNSCCUP is not only limited to identifying the primary tumour—but equally important—for the assessment of other nodal features, e.g., **e**xtra**n**odal tumour **e**xtension (ENE)—one of the most important factors negatively impacting survival. Although microscopic ENE cannot be detected by MRI and CECT, clearly positive imaging findings are reliable [9]. The 9th AJCC/UICC TNM classification has now incorporated ENE detected at cross-sectional imaging (i-ENE) as a criterion for N classification [7].

Several authors have reported a higher detection rate of the HNSCCUP with FDG PET CT compared to CECT/MRI, however, also higher false-positive rates due to physiologic uptake in lymphatic structures or due to infection/chronic inflammation [1, 3]. Conversely, other authors reported limited incremental benefit of FDG PET CT over physical exam and CT/MRI, also noting high false-positive rates in the oropharynx [4]. More recently, clinical exams combined with tonsillectomy and panendoscopy were found to be more effective than imaging, with PET CT not outperforming CT/MRI [10]. PET CT also appears to be less effective in identifying the primary tumour in HPV-negative compared to HPV-positive cases [2]. While the benefit of FDG PET CT in primary tumour localisation remains debated, it should be performed in patients with “MRI occult tumours” and to detect additional disease sites affecting treatment. Performing FDG PET CT prior to invasive biopsies is advised to reduce false positives from post-procedural inflammation. Based on the available sparse literature, FDG PET MRI appears to have comparable diagnostic accuracy to FDG PET CT. Therefore, the role of FDG PET MRI in HNSCCUP currently remains unclear despite theoretical benefits due to superior soft tissue evaluation [11]. Recently, FAPI PET CT, unlike FDG PET CT, has shown promise in detecting HNSCCUP, particularly when FDG PET CT is inconclusive, by offering higher sensitivity and better tumour-to-background contrast [12]. FAPI PET CT utilises ^68^Ga that binds to the fibroblast activation protein (FAP), which is expressed in many cancers, including HNSCC.

## Opportunities and challenges for the multidisciplinary clinical team

Despite exhaustive diagnostic protocols, the primary tumour is detected in only up to 60% of HNSCCUP cases [1–3, 8, 10]. This reflects the fundamental biological complexity of these cancers. Although therapeutic decisions are usually taken by multidisciplinary tumour board teams, treatment of HNSCCUP varies as current guidelines do not provide clear recommendations regarding the most effective treatment, and several issues are unresolved. For example, the benefits of neck dissection (ND) and superselective ND in HNSCCUP are unclear. Likewise, aggressive treatment with total mucosal irradiation is a controversial topic with potential benefits and drawbacks. This variability in treatment partly explains the wide range of reported 5-year overall survival rates, between 49% and 73% [1–3, 8, 10]. Factors negatively influencing the outcome of patients with HNSCCUP include higher N categories, HPV-negativity, and surgery alone compared to surgery with adjuvant irradiation in unilateral disease [2].

## Recommendations for clinical practice

Key recommendations for clinical practice include:

- Initial thorough physical examination and flexible fibreoptic endoscopy, if possible, with NBI
- US and USFNAC for accurate diagnosis of nodal metastases, with concurrent HPV/EBV testing; USFNAC can then be complemented by core needle biopsy in inconclusive USFNAC and to obtain material for additional biomarkers, e.g., PD-L1.
- Multiparametric MRI (with DWI) or CECT (if MRI is not feasible) for detailed anatomical assessment, TNM classification, surgical planning and for evaluating anatomical variants if considering TORS
- FDG PET CT if the tumour is “MRI occult” and to detect additional disease sites affecting treatment
- Panendoscopy (with NBI) under general anaesthesia, with directed biopsies of suspicious mucosal sites, bilateral tonsillectomy and base of tongue mucosectomy
- Multidisciplinary case discussion by the tumour board team consisting of specialised head and neck surgeons, oncologists, radiotherapists, radiologists, pathologists and other disciplines; clinical, radiological, histological and molecular findings are then integrated to facilitate consensus regarding diagnosis and individualised treatment planning

These recommendations aim to maximise primary tumour detection, enable precision treatment, reduce morbidity, and improve overall outcomes for patients with HNSCCUP. Yet guidelines continue to evolve as new evidence and technologies emerge.

## Directions for future research

Directions for future research focus on advancing diagnostic imaging and biopsy techniques to enhance primary tumour localisation as a clinical imperative in the management of HNSCCUP. High-resolution multiparametric MRI (with DWI and perfusion imaging) and PET CT with novel tracers, such as FAPI, may surpass FDG PET CT in sensitivity for detecting hidden lesions. Larger studies on their use in patients with HNSCCUP are needed. Future studies should also establish standardised, multimodal imaging protocols combining structural, functional, and molecular imaging to streamline diagnostic pathways. The use of decision support systems and image-based predictive models may further improve accuracy, reduce unnecessary procedures, and optimise personalised treatment planning for HNSCCUP patients. De-escalation trials, integrating HPV status and treatment response, are necessary to explore reduced radiation doses and the role of immunotherapy or targeted agents. Multi-institutional registries and standardised protocols will be essential to unify research and advance HNSCCUP care globally.
